# The myth of iodine: A systematic review and meta-analysis on the relationship between iodine and thyroid nodule

**DOI:** 10.1007/s40618-025-02606-4

**Published:** 2025-05-28

**Authors:** Willy Gräfe, Sandy Scheibe, Josy Schwarz, Lukas Liebig, Karen Voigt, Jeannine Schübel

**Affiliations:** Department of General Practice, Faculty of Medicine Carl Gustav Carus, Dresden University of Technology, Dresden, Saxony Germany

**Keywords:** Thyroid nodule, Systematic review, Meta-analysis, Iodine intake, Urinary iodine concentration

## Abstract

**Background:**

Iodine is an essential trace element for thyroid hormone synthesis, and its role in thyroid health has been widely studied. While iodine deficiency is recognized as a risk factor for goiter, its association with thyroid nodules remains controversial. The aim of this systematic review and meta-analysis was to evaluate the relationship between iodine intake and the development of thyroid nodules.

**Methods:**

A systematic literature search was conducted in Medline (via PubMed), the Cochrane Library, and guideline registries (AWMF, GIN) for studies published between 2012 and 2023. Inclusion criteria focused on studies examining the association between iodine intake and thyroid nodule. Systematic review has been conducted whereas study quality was assessed using the checklists of Critical Appraisal Skills Programme (CASP). A meta-analysis was performed for studies reporting odds ratios based on WHO-defined iodine categories.

**Results:**

A total of 31 studies were included. Most studies (n = 23) were cross-sectional, limiting causal conclusions. The most used method for assessing iodine intake was urinary iodine concentration (UIC), though measurement approaches varied. N = 10 studies compared median UIC between groups with and without thyroid nodules, with n = 8 reporting significant differences. However, the iodine levels in both groups often remained within the WHO-defined adequate iodine range. N = 8 studies examined odds ratios for iodine intake and thyroid nodule risk, with n = 5 identifying iodine deficiency (< 100 μg/L) as a significant risk factor. However, results for more than adequate (> 200 μg/L) and excessive iodine intake (> 300 μg/L) were inconsistent. N = 3 studies suggested a U-shaped relationship between iodine and thyroid nodule prevalence, but meta-analysis findings did not confirm this hypothesis. The pooled odds ratio for iodine deficiency was 1.24 (95% CI [1.16–1.33], I^2^ = 0.00), while more than adequate and excessive iodine intake showed no significant association.

**Conclusion:**

This systematic review and meta-analysis indicate that iodine deficiency increases moderately the risk of developing thyroid nodules, while more than adequate and excessive iodine intake does not show a consistent effect. However, the heterogeneity of study results and the predominance of cross-sectional designs limit definitive conclusions. Further prospective studies are needed to clarify the causal relationship between iodine intake and thyroid nodules.

**Supplementary Information:**

The online version contains supplementary material available at 10.1007/s40618-025-02606-4.

## Introduction

Iodine is an important trace element for the formation of thyroid hormones [1, 2]. In the past, iodine was primarily attributed a role in the synthesis of thyroid hormones, in particular thyroxine (T4) and triiodothyronine (T3) [3], by the thyroid gland [1, 4]. Recent experimental studies (in vitro) suggest that iodine may also activate antiproliferative and apoptotic effects in malignant cells [5]. Defined by WHO, the optimal iodine intake for people over an age of 12 years is 150 μg/day [6, 7]. With a median iodine excretion of 100–299 μg/L via the urine, iodine intake is ideal [5].

In adults, mild to moderate iodine deficiency appears to be associated with a higher incidence of more aggressive subtypes of thyroid carcinoma (anaplastic and follicular thyroid carcinoma), an increased risk of diffuse strands, an increased risk of non-toxic and toxic nodular goitre and hyperthyroidism or hypothyroidism [1, 8]. In the late 1980 s, the European Thyroid Association re-evaluated the problem of iodine deficiency. With the exception of the Scandinavian countries, Austria and Switzerland, most European countries were still affected by iodine deficiency in the late 1980 s [4]. In 1993, the WHO and UNICEF recommended the widespread iodisation of table salt as the main strategy to counteract iodine deficiency disorder (IDD). Since 1990, the iodisation of table salt has been significantly promoted worldwide [9], enabling many countries to achieve or come close to the goal of reducing IDDs [6]. Nevertheless, in 2007 around 31% of the world's population and 52% of the European population were still affected by insufficient iodine intake [6]. The WHO defines criteria for iodine supply at population level. Insufficient iodine supply is defined as an iodine concentration in urine of < 100 μg/L; adequate iodine supply 100–199 μg/L; more than adequate iodine supply 200–299 μg/L and excessive iodine supply > 299 μg/L [10, 11]. At population level, no more than 20% of a population should have an iodine concentration of less than 50 μg iodine per litre of urine [6].

It is often assumed that iodine plays a decisive role in the development of thyroid nodules and that iodine deficiency is the most important established risk factor for the development of nodular thyroid diseases [12] or that thyroid nodules are more prevalent in iodine-deficient regions [13–15]. Pathogenetically, iodine deficiency is also regarded as a growth promoter that makes the mutations on which the development of thyroid nodules is based more likely [1]. However, it remains unclear whether iodine deficiency directly causes the development of thyroid nodules or whether other factors, such as genetic predisposition or environmental conditions, also play a role. While epidemiological data suggest an association, a definitive experimental proof of a causal relationship is still lacking.

A thyroid nodule is defined as a discrete lesion of the thyroid gland that can be differentiated radiologically from the surrounding parenchyma of the thyroid gland [16]. Most patients affected by thyroid nodules are asymptomatic. If symptoms such as globus sensation, dysphagia or dyspnoea occur, these are usually due to compression of surrounding structures by the nodule [17]. It is particularly important to rule out malignancy of the nodule, which can occur, based on a recent study, in 1.1% of cases [18]. The prevalence of thyroid nodules varies with the method of examination, gender, age and the region in which it is performed [19–21]. It is therefore difficult to state the exact prevalence of thyroid nodules in a population. A Korean study found a prevalence of 34% in their study population [19]. In China, 43% [21] and in Germany, 68% [20]. It is questionable to what extent these prevalence rates can be applied to the respective total population.

There are no systematic or controlled studies that prove that iodine deficiency is the cause of thyroid nodules. Previous reviews were unsystematic and often only considered the relationship between iodine and thyroid nodules alongside other endpoints. The aim of this systematic review was therefore to systematically analyse the relationship between iodine and the development of thyroid nodules based on published studies and guidelines.

## Methods

The systematic review was conducted following the Preferred Reporting Items for Systematic Reviews and meta-analysis (PRISMA) [22].

### Literature research

We searched the medical databases Medline (via Pubmed) and Cochrane Library as well as the AWMF and GIN guideline registers for relevant publications. In addition, we conducted a comprehensive hand search (reverse search) to identify further suitable publications for the research project. The systematically developed search strategy was based on the PICO(S) scheme. Due to the research question, only keywords/MESHs in the categories population, intervention and outcome were considered.

### Inclusion and exclusion criteria

We selected relevant MESH-Terms for the categories based on thematically related systematic reviews [3, 23–25]. We only considered literature with a publication date between 1 January 2012 and 30 June 2023 in order to include only current evidence and included RCT, cohort studies, cross-sectional studies and case–control studies. We excluded publications that treated pregnant women, children and adolescents (< 18 years). We also excluded case reports and case series as well as articles that were not written in German or English.

### Study selection

We chose a two-stage screening procedure to select the studies. First, the titles and abstracts were screened by only one reviewer due to limited human resources. In case of uncertainty, the reviewer consulted a second person and the two of them made the decision to include or exclude the study. Second, two reviewers independently screened all full-text articles that at least one of them considered potentially relevant after applying the inclusion and exclusion criteria. Studies that were assessed as irrelevant were documented with a corresponding reason for exclusion. The reviewers reached a consensus together in the event of disagreements and, if necessary, consulted a third person of the research team to reach a decision.

### Data extraction

Data extraction was carried out independently by two reviewers using a predefined extraction table. In addition to metadata of the studies (author, year of publication, country), they extracted information on the study population, analysed endpoints, iodine measurement methods and the main results.

### Evaluation of study quality

Two reviewers used CASP (Critical Appraisal Skills Programme) checklists to assess the methodological quality of the articles. CASP is a toolset for the structured assessment of scientific studies and includes questions on the relevance of the study, recruitment or transferability of the study [26].

### Statistical analysis

The extracted data from identified primary studies were checked for clinical, methodological and statistical comparability and a decision was then made on whether to conduct a meta-analysis [27]. We calculated random-effects meta-analysis and selected publications that recorded the association between iodine and thyroid nodules as odds ratios according to the WHO criteria for iodine supply. A funnel plot analysed a possible publication bias. The I^2^ statistic describes the heterogeneity between the studies. Low, medium and high heterogeneity are defined by I^2^ values of 25%, 50% and 75%, respectively. We calculated the meta-analysis with SPSS version 30.0.

### Utilisation of AI

We used ChatGPT 4.5 as a writing aid for this publication. Complex sentence structures were simplified with this AI. DeepL was used for the translation of the publication from German into English.

## Results

### Study selection

The search in the Medline database (via Pubmed) took place on 13 July 2023 and yielded a total of n = 758 potentially relevant articles. The search in the Cochrane database on 25 September 2023 yielded a total of n = 67 potentially relevant articles. These included n = 2 duplicates. The GIN and AWMF guideline registers did not contain any papers relevant to the research project.

**Fig. 1:**
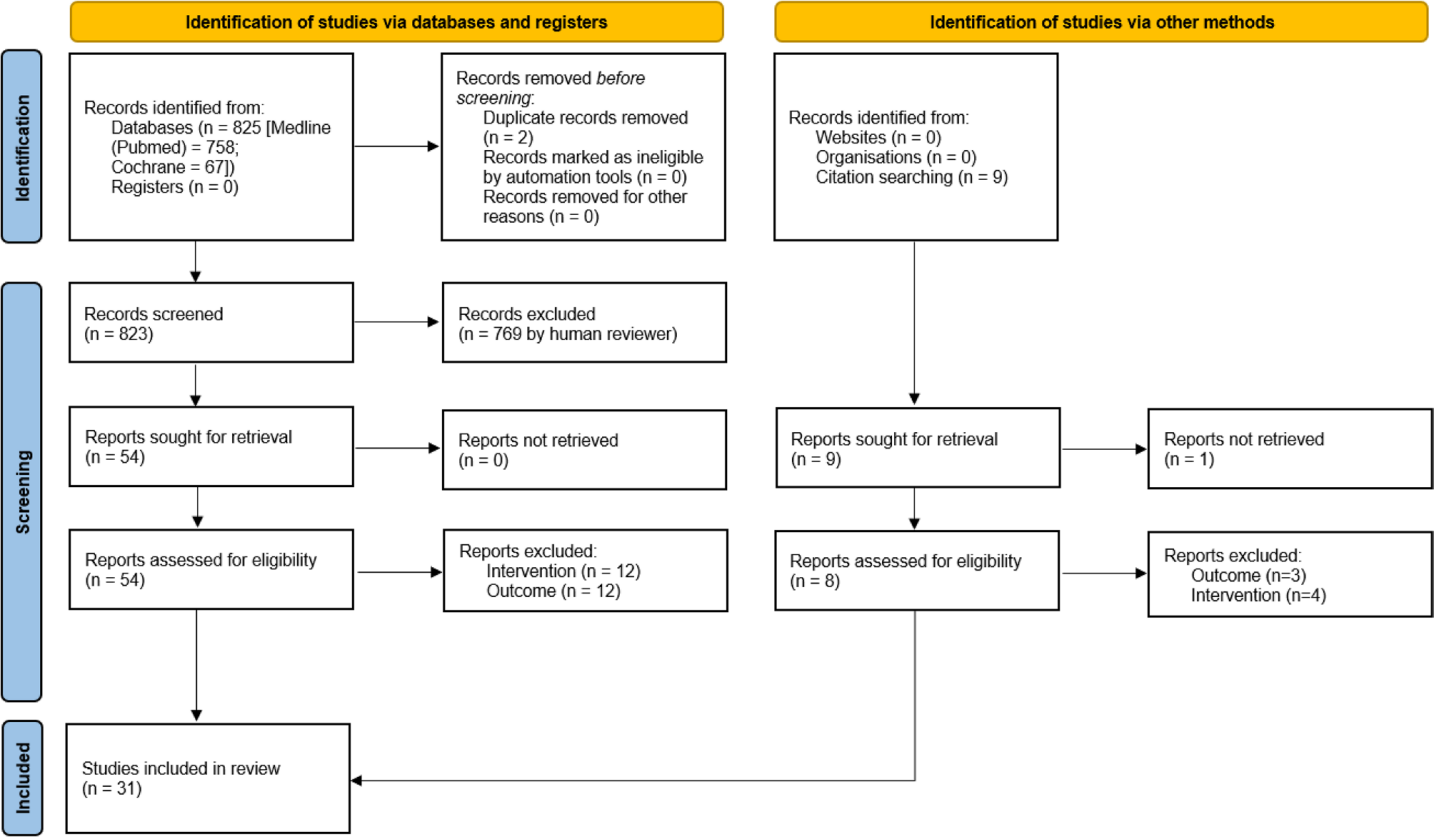
PRISMA flowchart of study search and selection pathway

The hand search yielded n = 9 further articles, meaning that we examined a total of n = 832 papers in the course of the title-abstract screening. A total of n = 54 articles remained for the subsequent full-text screening. In the course of the full-text screening, the reviewers excluded a total of n = 23 articles for objective reasons, including n = 12 articles due to the intervention and n = 11 articles due to the outcome investigated in the studies. The excluded studies did not make any statements or calculations on the relationship between iodine concentration and nodules. The systematic search yielded a total of n = 31 articles for the evidence synthesis, including n = 26 studies [14, 28–52] and n = 5 evidence syntheses [53–57]. The selection of articles is shown in Fig. 1.

Two reviewers independently assessed the study quality using the CASP criteria. They were unable to rule out potential risks of selection bias in n = 8 studies. The reviewers identified the following reasons: a missing random sample, undescribed recruitment, a very small number of cases or the restriction of the sample to patients from a single hospital [31, 37, 42, 44, 47–50].

### Study characteristics of the primary studies

The total number of individuals in the included studies was n = 9 575 297 (median = 2678.5; range: 62 to 9 381 032) with an age range of 6 to 85. The more detailed descriptions of the study populations are provided in Table 1. Of the n = 31 included articles, n = 27 were conducted in China [14, 28–30, 32–36, 38–52, 55–57], the remaining in Denmark [53], Israel [37], Switzerland/UK [54] und Cyprus/Romania [31]. N = 23 articles were cross-sectional studies [14, 28–30, 32–38, 40–48, 50–52], n = 2 were case control studies [31, 49], n = 1 article was a cohort study [39]. We were also able to include n = 3 systematic reviews including meta-analysis [55–57] and n = 2 unsystematic reviews [53, 54]. The detailed study characteristics are shown in Table 2. The systematic search did not reveal any clinical study that causally investigated the use of iodine for the reduction and/or prevention of thyroid nodules.

**Table 1:** Study characteristics of the primary studies

First author (year, country)	Study type	n	Study population
Chen (2013, China) [28]	Cross-sectional study	9412	Age > 5 years, resident of Hangzhou province, Zhejiang in eastern China
Du (2014, China) [29]	Cross-sectional study	2147	Adults > 17 years, residents of the villages Fengrun, Yangfang, Dongwenzhuang, Caizhuang (high iodine concentration in water); villages Shuangzhai, Houhuangtai, Lizhuang, Anrong, Hutong, Xicuan (sufficient iodine concentration); Xicuan, Guangxi (low iodine concentration), China
Fan (2018, China) [30]	Cross-sectional study	2647	Subjects > 18 years, belonging to Han Chinese (ethnic group)
Gaengler (2017, Cyprus/Romania) [31]	Case control study	208	Women > 18 years, resident in Cyprus and Romania
Gu (2016, China) [32]	Cross-sectional study	17,056	Age > 6 years, resident of Zhejiang province, China
Guo (2016, China) [33]	Cross-sectional study	1591	Adults between the ages of 18 and 84, residents of Xinjiang Province (China)
Li (2020, China) [34]	Cross-sectional study	78,470	Adults (> 18 years) from 31 provinces in China
Lou (2020, China) [35]	Cross-sectional study	2710	Subjects from the Zhejiang Province of China (eastern coast of China), age > 18 years
Miao (2020, China) [36]	Cross-sectional study	3754	Adults (20–70 years) from Heilongjiang Province in China
Ovadia (2014, Israel) [37]	Cross-sectional study	62	Subjects between 21–80 years old, voluntary sample, living in Ashkelon (Israel)
Qu (2022, China) [38]	Cross-sectional study	12,271	2009 study: residents from the general population of China (15–69 years) who have lived in the region for at least 6 months, 2016/2017 study: test persons who have lived in Shanghai for at least 6 months, age 18–65 years, test persons were matched for the study: 3,474 couples, age 18–65 years
Shan (2016, China) [40]	Cross-sectional study	15,008	Age > 15 years Residents from 10 cities in China (East and Central China)
Shan (2023, China) [39]	Cohort study	8868	Population from three regions of China: Panshan (iodine deficiency area), Zhangwu (area with more than sufficient iodine intake), Huanghua (area with excessive iodine intake); age 14–73 years
Shao (2016, China) [41]	Cross-sectional study	835	Age between 25–65 years, resident of Huan-cui district in Shandong province (China)
Song (2016, China) [14]	Cross-sectional study	5144	People aged 15–69, residents of the city of Shanghai, China
Sun (2020, China) [43]	Cross-sectional	12,98	Subjects > 15 years, residents from 10 cities in East & Central China
Tian (2021, China) [42]	Cross-sectional study	3661	Adults aged 20–70, residents of Heilongjiang Province, China
Wang (2014, China) [44]	Cross-sectional study	460	Adults from China
Yan (2021, China) [46]	Cross-sectional study	2516	Age > 18 years, resident of the city of Guangzhou, China
Yan (2023, China) [45]	Cross-sectional study	2636	Subjects > 18 years, belonging to Han Chinese (ethnic group)
Yao (2022, China) [47]	Cross-sectional study	1344	Age between 18—60 years, inhabitants of the villages Xieyuanji and Jiucheng in Heze, China (areas with excessive iodine levels), villages Donding in Jining and Yangying in Heze, China (areas with adequate iodine levels), Wanfang in Heze (iodine deficiency area)
Yu (2021, China) [48]	Cross-sectional study	1341	Age > 18 years, resident of Shaanxi province in China
Yuan (2023, China) [49]	Case control study	345	Age between 18 and 70, resident of China
Zhao (2014a, China) [50]	Cross-sectional study	1177	Patients with and without thyroid disease from southern China
Zhu (2022, China) [51]	Cross-sectional study	9,381,032	Adults (> 18 years in 2018) living in 30 municipalities and provinces in China
Zou (2012, China) [52]	Cross-sectional study	7904	All residents of the city of Shanghai (China) (living in the region for at least 12 years), aged between 5–69 years

**Table 2:** Study characteristics of the meta-analysis and reviews

First author (year, country)	Study type	Number of studies n	Characteristics of the included studies	Main results
Carlé (2014, Denmark) [53]	Unsystematic review	Not named	Not named	Iodine deficiency is the cause of thyroid nodules
Liu (2021, China) [57]	Meta-analysis	34	Studies had to be population-based, include community-oriented random samples (not hospital-based or voluntary participants), and provide sufficient research details (e.g., geographic region, methodology, sample size, diagnostic criteria, and urinary iodine concentration)	Pooled prevalence of thyroid nodules: Low iodine intake (< 100 µg/L) 20.7% Adequate iodine intake (100–199 µg/L) 27.2% More than sufficient iodine intake (100–299 µg/L) 16.0% Excessive iodine intake (≥ 300 µg/L) 18.9%
Weng (2017, China) [56]	Meta-analysis	43	Studies had to be population-based, include random community-based samples (not hospital-based or voluntary participants), and provide sufficient outcome details (e.g., survey location, methodology, diagnostic criteria, sample size, and urinary iodine concentration)	Pooled prevalence of thyroid nodules: Low iodine intake (< 100 µg/L) 22.3% Adequate iodine intake (100–299 µg/L) 25.4% Excessive iodine intake (≥ 300 µg/L) 6.8% Pooled prevalence of excessive iodine intake differed from the other two groups (p < 0.01)
Zhao (2014b, China) [55]	Meta-analysis	52	Studies had to be population-based (not hospital-based), include randomly selected community samples from mainland China, provide sufficient data for pooled analysis	Increase in prevalence of thyroid nodules from 11% to 24.4% in China after 2002
Zimmermann (2015, Switzerland/Great Britain) [54]	Unsystematic review	Not named	Not named	Iodine deficiency as well as iodine excess is the cause of thyroid nodules Risk of developing nodules 25–26% higher for people who do not consume iodised salt

### Measurement of iodine

Iodine status was determined in n = 24 of 26 studies by measuring the iodine concentration in spontaneous urine (UIC) in micrograms per litre (μg/L) after a fasting period [14, 28–36, 38–50, 52]. Other measurement methods include measuring the iodine concentration in blood, drinking water, table salt or soil or enquiring about iodine consumption using a dietary questionnaire [14, 28, 29, 32, 34, 35, 37–39, 43, 45–47, 51, 52].

### Connection between iodine and thyroid nodules

The detailed results of the respective studies are shown in Table 3. The study situation is heterogeneous, but some results can be summarised.

**Table 3:** Main results of the primary studies

First author (year, country)	Measurement iodine	Main results
Chen (2013, China) [28]	Urinary Iodine Concentration (UIC) Nutrition questionnaire Iodine concentration in table salt and drinking water	Reference category = Sufficient iodine intake (100–199 µg/L) Iodine deficiency (< 100 µg/L) positively associated with nodules (OR 1.27 [CI 1.07–1.45], p = 0.004) More than adequate iodine intake (200–299 µg/L) (OR = 1.01 [CI 0.86–1.18], p = 0.893) Excessive iodine intake (≥ 300 µg/L) (OR 0.97 [CI 0.82–1.14], p = 0.682) Adults who consumed non-iodised salt have an increased risk of thyroid nodules (OR: 1.36, [CI 1.01–1.83], p = 0.041)
Du (2014, China) [29]	Nutrition questionnaires Iodine concentration in table salt and drinking water UIC	Prevalence of thyroid nodules differed significantly between the three groups with different iodine levels (× 2 = 39.779, p = 0), between the iodine deficient group and the iodine sufficient group (x^2^ = 39.234, p = 0), between the group with more than adequate iodine supply and the group with adequate iodine (x^2^ = 13.964, p = 0) and between the group with more than adequate iodine supply and the group with iodine deficiency (x^2^ = 11.125, p = 0.001) Reference category = Sufficient iodine intake (100–199 µg/L) Iodine deficiency (< 100 µg/L) positively associated with nodules (OR 2.97 [CI 2.05–4.32], p < 0.01) Excessive iodine intake (≥ 400 µg/L) (OR = 1.87 [CI 1.30–2.68], p < 0.01)
Fan (2018, China) [30]	UIC	Prevalence of nodules differed significantly between groups with different iodine levels; U-curve was confirmed: Prevalence of 25.3% in the group of subjects with 100–300 ug/L UIC, prevalence decreased significantly with iodine concentration > 150 ug/L. Subjects who consumed iodised salt had a lower prevalence of thyroid nodules than subjects who did not consume iodised salt (25.4% vs. 29.8%, x^2^ = 4.384 p = 0.036) Association between nodules and iodine concentration in urine could not be shown in the regression analysis
Gaengler (2017, Cyprus/Romania) [31]	UIC	Reference category = Sufficient iodine intake (100–199 µg/L) Women in iodine deficiency areas (< 100 µg/L) (OR 1.29, [CI 0.69–2.41], p = 0.427) Women in areas with excessive iodine intake (≥ 200 µg/L) (OR 1.39, [CI 0.65–3.01], p = 0.394)
Gu (2016, China) [32]	Iodine concentration in table salt and drinking water UIC questionnaire	Median iodine concentration in urine: group with thyroid nodules vs. group without thyroid nodules 154.0 µg/L vs. 165.0 µg/L p < 0.001 Proportion of people with nodules who did not consume iodised salt was significantly higher than the proportion of people who consumed iodised salt (30.1% vs. 19.2%, p < 0.001)
Guo (2016, China) [33]	UIC	Prevalence of nodules in the iodine-deficient group: 27.2%; adequate iodine supplementation group: 28.5%, excessive iodine supplementation group: 25.6%; no significant difference in prevalence between the three groups (χ2 = 0.705, P = 0.703)
Li (2020, China) [34]	Nutrition questionnaire UIC	Reference category = Sufficient iodine intake (100–199 µg/L) Iodine deficiency (< 100 µg/L) positively associated with nodules (OR 1.27 [CI 1.19–1.37], p < 0.0001), More than adequate iodine intake (200–299 µg/L) lower risk of nodules (OR 0.88 [CI 0.80–0.97], p = 0.01) Excessive iodine intake (≥ 300 µg/L) lower risk of nodules (OR = 0.74 [CI 0.65–0.85], p = 0.0001)
Lou (2020, China) [35]	Nutrition questionnaire UIC	Median iodine concentration in urine: group with thyroid nodules vs. group without thyroid nodules 122.9 µg/L vs. 164.0 µg/L p < 0.01 Correlation coefficient for salt consumption and median iodine concentration = 0.4 (p < 0.01) Subjects who did not consume iodised table salt had a significantly higher prevalence of nodules (p < 0.01) Significant quadratic and linear correlations were found between the concentration of iodine in urine and the prevalence of thyroid nodules (p < 0.01) highest prevalence of nodules in the group of subjects with iodine deficiency Reference category = iodine deficiency (< 100 µg/L) Sufficient iodine intake (100–199 µg/L) (OR 0.75 [CI 0.58–0.97], p = 0.03), More than adequate iodine intake (200–299 µg/L) (OR 0.54 [CI 0.39–0.75], p = 0.000) Excessive iodine intake (≥ 300 µg/L) lower risk for nodules (OR 0.50 [CI 0.34–0.76], p = 0.000) 69–77% risk reduction for nodules when taking iodised salt
Miao (2020, China) [36]	UIC Salt sample	Prevalence of focal thyroid lesions depending on iodine intake (low, sufficient, more than sufficient): 34.86%, 36.72%, 37.60%
Ovadia (2014, Israel) [37]	Nutrition questionnaire	At 65 ± 30 g/day, the mean estimated iodine intake of the group with nodular goitre was 42% lower than that of the control group (115 ± 60 g/day) (p < 0.05)
Qu (2022, China) [38]	Nutrition questionnaire UIC	Reference category = iodine deficiency (< 100 µg/L) Sufficient iodine intake (100–199 µg/L) (OR 0.88 [CI 0.76–1.01], p = 0.06), More than adequate iodine intake (200–299 µg/L) (OR 0.96 [CI 0.81–1.14], p = 0.63) Excessive iodine intake (≥ 300 µg/L) lower risk of nodules (OR 0.85 [CI 0.68–1.05], p = 0.13)
Shan (2016, China) [40]	UIC	The prevalence of nodules was 14.5% in cities with sufficient iodine supply and 10.4% in cities with more than sufficient iodine supply (p < 0.001) The prevalence of nodules increased significantly in East and Central China between 1999 and 2011 (2.73% to 12.8%, p < 0.001)
Shan (2023, China) [39]	Oral questioning UIC	The incidence density for thyroid nodules (17.72 vs. 23.70 per 1000 person-years, p = 0.02) increased in the period 2004–2019 compared to 1999–2004. The Zhangwu region developed from more than sufficient iodine intake to sufficient iodine intake The incidence density for thyroid nodules (17.26 vs. 28.25 per 1000 person-years, p < 0.001) increased in the period 2004–2019 compared to 1999–2004. The Huanghua region developed from excessive iodine intake to more than sufficient iodine intake
Shao (2016, China) [41]	UIC	Median iodine concentration in urine: group with thyroid nodules vs. group without thyroid nodules 139.4 µg/L vs. 101.5 µg/L p < 0.01
Song (2016, China) [14]	Nutrition questionnaire UIC	Median iodine concentration in urine: group with thyroid nodules vs. group without thyroid nodules 143.1 µg/L vs. 135.4 µg/L p = 0.004 Median iodine concentration in women without nodules higher than in the group with nodules 139.01 µg/L vs. 129.32 µg/L p = 0.007 Median urinary iodine concentration in the group without nodules with iodised salt intake was higher than in the group with nodules (p = 0.003) U-curve relationship between urinary iodine concentration and prevalence of thyroid nodules with inflection point at 301 µg/L The prevalence of nodules decreased in women from 43.41% to 30.54% (iodine concentration < 150 µg/L), increased from 23.53% to 41.18% (iodine concentration from 301 µg/L to 500 µg/L). In men, no statistically significant difference was found in the iodine concentration and the prevalence of nodules
Sun (2020, China) [43]	Nutrition questionnaire UIC	U-curve for men with"turning point"at a concentration of 527 μg/L. negative, significant trend for 0–527 μg/L (adjusted OR = 0.87, [0.80–0.94], p < 0.001) positive (but non-significant) trend for UIC > 527 μg/L (adjusted OR 1.25, [0.98–1.60], p = 0.076) linear negative correlation for women (OR 0.95, [0.91—0.99], p = 0.023)
Tian (2021, China) [42]	Salt samples UIC	Median urinary iodine concentration: group with thyroid nodules vs. group without thyroid nodules (166.76 μg/L vs. 154.62 μg/L, p = 0.166)
Wang (2014, China) [44]	UIC	Median iodine concentration in urine: group with thyroid nodules vs. group without thyroid nodules 331.3 µg/L vs. 174.3 µg/L p < 0.001
Yan (2021, China) [46]	Nutrition questionnaire UIC	Median iodine concentration in urine: group with thyroid nodules vs. group without thyroid nodules 123.6 µg/L vs. 133.9 µg/L p < 0.001 Reference category = Sufficient iodine supply (100–199 µg/L) Iodine deficiency (< 100 µg/L) (OR 1.37 [CI 0.93–1.95], p = 0.113), More than sufficient iodine intake (200–299 µg/L) (OR 1.70 [CI 1.17–1.248], p = 0.006) Excessive iodine intake (≥ 300 µg/L) (OR: 1.20 [CI 0.80—1.80], p = 0.38)
Yan (2023, China) [45]	Nutrition questionnaire UIC	Reference category = adequate iodine supply (100–199 µg/L) Iodine deficiency (< 100 µg/L) (OR 1.22 [CI 0.67–2.20], p = 0.51), More than sufficient iodine intake (200–299 µg/L) (OR 1.27 [CI 0.73–2.24], p = 0.39) Excessive iodine intake (≥ 300 µg/L) (OR: 3.27 [CI 1.03—6.75], p = 0.03)
Yao (2022, China) [47]	Nutrition questionnaire UIC Iodine concentration in blood, table salt and drinking water	Reference category = urinary iodine concentration of 100–300 μg/L Iodine deficiency (< 100 μg/L) higher prevalence of nodules (37.17% vs. 21.14%, P < 0.05) Excessively high urinary iodine concentration (≥ 800 μg/L) higher prevalence of nodules (33.75% vs. 21.14%, P < 0.05) After controlling for age, gender and BMI, both excessively high urinary iodine concentration (OR 1.86 [CI 1.10–3.12]) and iodine deficiency (OR 2.08 [CI 1.46–3.49]) were risk factors for nodules After controlling for age, gender and BMI, serum concentration of iodine was not a risk factor for nodules
Yu (2021, China) [48]	UIC	Reference category = adequate iodine supply (100–199 µg/L) Iodine deficiency (< 100 µg/L) (OR 0.62 [CI 0.31–1.22], p = 0.61), More than sufficient iodine intake (200–299 µg/L) (OR 1.00 [CI 0.59–1.70], p = 0.98) Excessive iodine intake (≥ 300 µg/L) (OR: 1.51 [CI 0.89—2.56], p = 0.12)
Yuan (2023, China) [49]	UIC	Median iodine concentration in urine: group with thyroid nodules vs. group without thyroid nodules 164.0 µg/L vs. 121.5 µg/L p < 0.05
Zhao (2014a, China) [50]	UIC	Median iodine concentration in urine: group with thyroid nodules vs. group without thyroid nodules 180.0 µg/L vs. 169.6 µg/L p = 0.012
Zhu (2022, China) [51]	Iodine concentration in groundwater and soil	Higher levels of iodine in drinking water is linearly associated with an increase in the prevalence of thyroid nodules with Pearson's correlation coefficient of 0.47 (men p < 0.01), 0.40 (women p = 0.03) and 0.46 (total p < 0.01) No correlation between iodine content in soil and thyroid nodules
Zou (2012, China) [52]	Nutrition questionnaire UIC Iodine concentration in table salt and drinking water	Median urinary iodine concentration: group with single thyroid nodules vs. group without thyroid nodules 139.5 µg/L vs. 143.3 µg/L not significant Median urinary iodine concentration: group with multiple thyroid nodules vs. group without thyroid nodules 129.3 µg/L vs. 143.3 µg/L not significant

### Mean differences: Comparison of median iodine concentration (MUI) between groups with and without thyroid nodules

N = 10 studies compared the median urinary iodine concentration (MUI) between the group with thyroid nodules and a control group without thyroid nodules. N = 8 studies found significant group differences [14, 32, 35, 41, 44, 46, 49, 50], while n = 2 studies found no significant group differences [42, 52]. In n = 9 studies [14, 32, 35, 41, 42, 46, 49, 50, 52] the MUI of the group with thyroid nodules was within the optimal iodine range defined by the WHO (100–199 μg/L) (Table 4).

**Table 4:** Differences in the median iodine concentration (in μg/L) in urine between the group without thyroid nodules (TN) and the group with at least one thyroid nodule (WHO defined optimal iodine range is 100–199 μg/L)

Source	Without TN (μg/L)	TN (μg/L)
Shao et al. (2016) [41]	101.5	139.4
Yuan et al. (2023) [49]	121.5	164.0
Yan et al. (2021) [46]	133.9	123.6
Song et al. (2016) [14]	143.1	135.4
Zou et al. (2012) [52]	143.3	139.5
Tian et al. (2021) [42]	154.6	166.8
Lou et al. (2020) [35]	164.0	122.9
Gu et al. (2016) [32]	165.0	154.0
Zhao et al. (2014) [50]	169.6	180.0
Wang et al. (2014) [44]	174.3	331.3

### Odds ratios: iodine intake and thyroid nodules

N = 8 studies were based on the WHO criteria to assess the iodine range in which the risk of developing thyroid nodules increases or decreases (odds ratios (OR)). N = 5 out of eight studies were able to identify iodine deficiency (< 100) as a significant risk factor for the development of thyroid nodules [28, 29, 31, 34, 47] while n = 3 out of eight studies were unable to identify a significant association between iodine deficiency and thyroid nodules [45, 46, 48]. A more than adequate iodine intake (200–299) was a risk factor in n = 1 study [46], and a protective factor in n = 2 studies [34, 35]. N = 4 studies were unable to identify a significant association [28, 31, 45, 48]. Excessive iodine intake (> 299 μg/L) was a risk factor in n = 3 studies [29, 45, 47], although according to the study by Yao et al. (2022), the risk of developing thyroid nodules was only increased from a MUI > 800 μg/L. The risk was not significantly increased in the 300 to 800 μg/L range [47]. N = 1 study found a risk reduction due to excessive iodine intake [34]. N = 4 other studies found no significant association between excessive iodine intake and the development of thyroid nodules [28, 31, 46, 48].

### Prevalences: relationship between iodine and the prevalence of thyroid nodules

N = 6 studies investigated the prevalence of thyroid nodules [33, 35, 36, 38, 47, 51]. N = 2 studies found no significant differences in the prevalences in the iodine sufficiency, deficiency and excess ranges [33, 36]. N = 2 studies showed that the highest prevalence of thyroid nodules was in the deficient range [35, 47]. N = 3 studies postulated a U-curve relationship between urinary iodine concentration and the prevalence of thyroid nodules [14, 30, 43], which means the prevalence of thyroid nodules decreased with increasing iodine concentration and increased again from an identified inflection point (150 μg/L [30]; women: 301 μg/L [14]; men: 527 μg/L) [43]).

### Consumption of iodised salt and thyroid nodules

N = 4 studies investigated the extent to which iodised salt consumption is associated with the development of thyroid nodules [28, 30, 32, 35]. The studies found a lower prevalence of thyroid nodules in the group that consumed iodised salt compared to the group that did not consume iodised salt [28, 30, 32, 35].

### Overview of reviews

The detailed results of the included meta-analysis and reviews are shown in Table 2.

The systematic review and meta-analysis by Weng et al. (2017) showed pooled prevalences of thyroid nodules for different groups: 22.3% in people with low iodine intake, 25.4% with sufficient iodine intake (here 100–299 μg/L) and 6.8% with excessive iodine intake. The prevalence in the group with excessive iodine intake differed significantly from the other groups (p < 0.01) [56].

The systematic review and meta-analysis by Liu et al. (2021) found the following pooled prevalences: 20.7% in people with low iodine intake, 27.2% with adequate iodine intake, 16.0% with more than adequate iodine intake and 18.9% with excessive iodine intake [57]. Zhao et al. (2014b) showed an increase in the prevalence of thyroid nodules from 11% to 24.4% in China after 2002.

The n = 2 unsystematic reviews both argue in favour that iodine deficiency stimulates nodule formation in the thyroid gland [53, 54]. Zimmermann and Boelaert (2015) add iodine excess as a further cause of thyroid nodules and conclude a U-shaped relationship between iodine and thyroid nodules [54].

### Meta-analysis

We included n = 7 studies in the meta-analysis [28, 34, 35, 38, 45, 46, 48]. These n = 7 studies all had odds ratios as effect estimates based on the criteria for iodine intake defined by the WHO [10, 11].

**Fig. 2:**
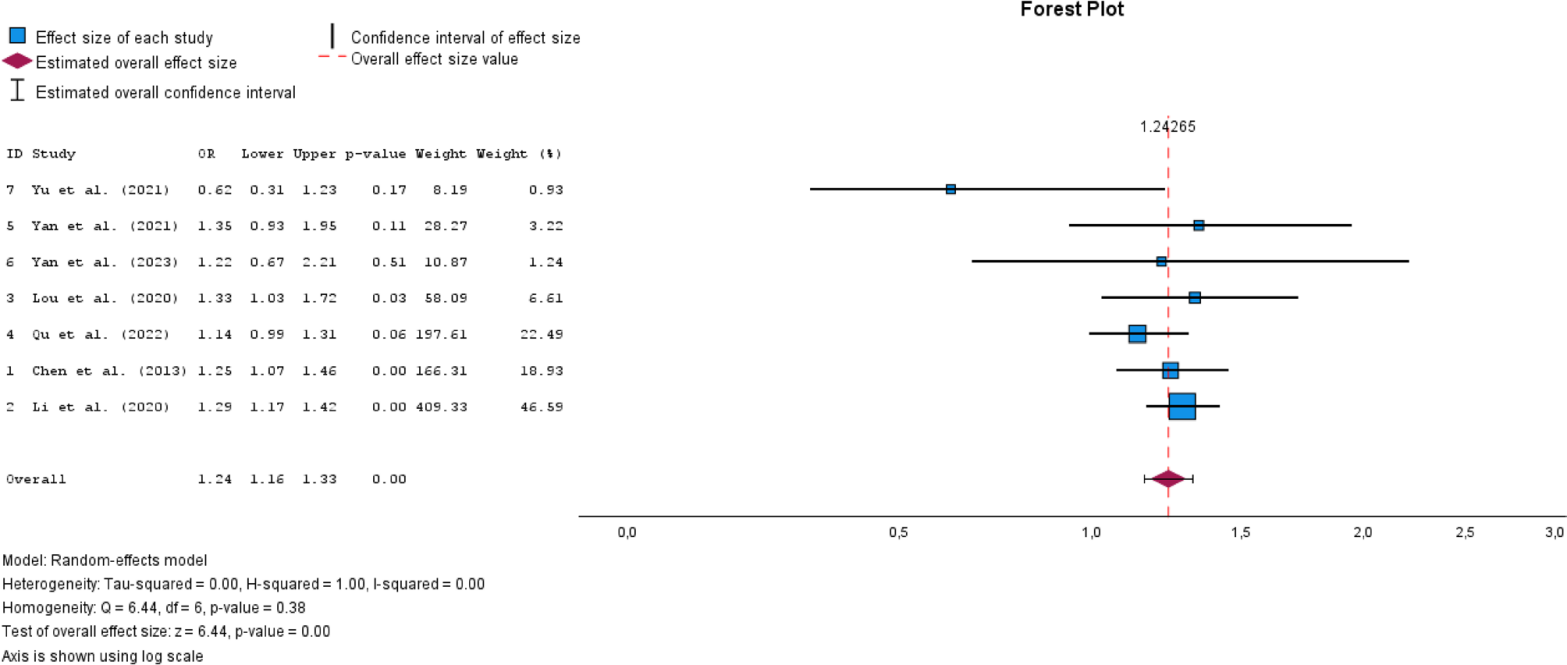
Forest plot pooled odds ratios iodine deficiency vs. sufficient iodine supply

**Fig. 3:**
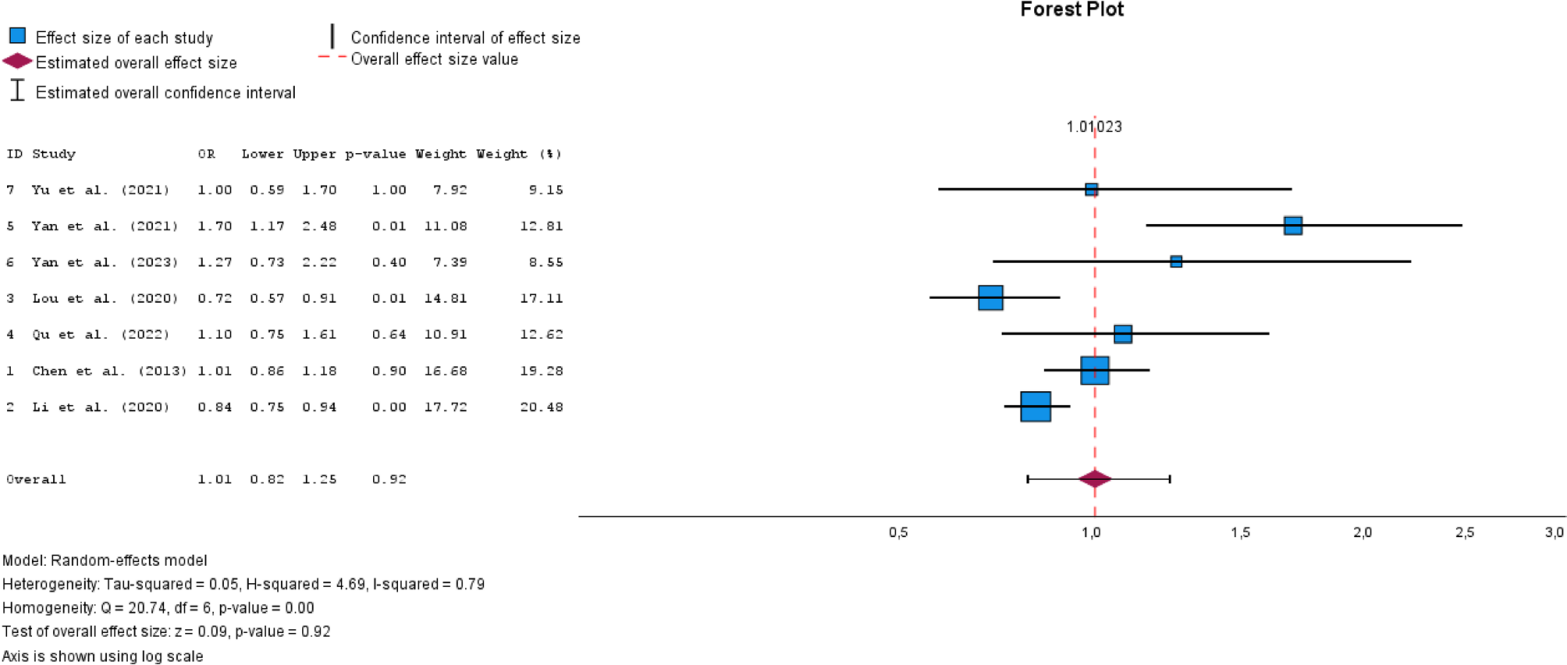
Forest plot more than sufficient iodine supply vs. sufficient iodine supply

**Fig. 4:**
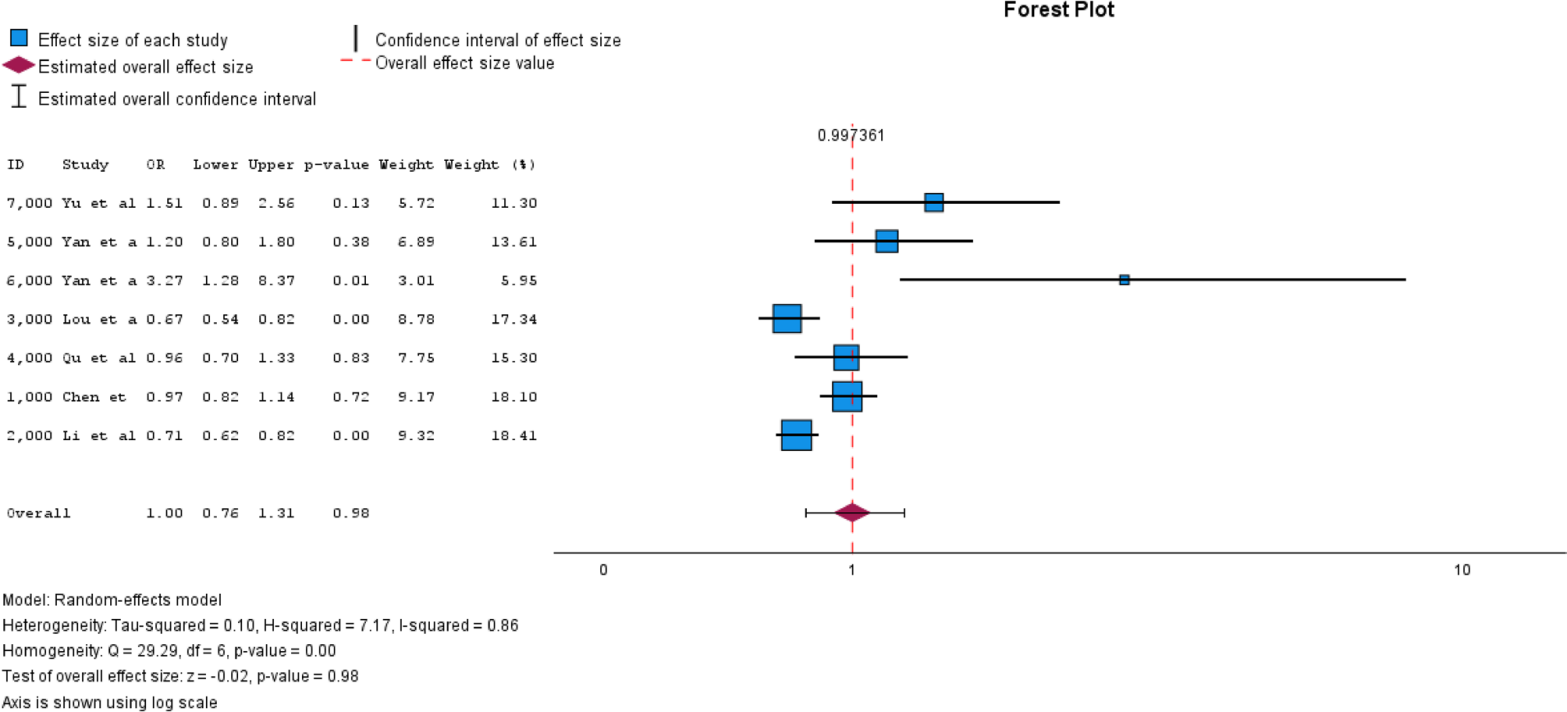
Forest plot excessive iodine supply vs. sufficient iodine supply

The reference category was adequate iodine supply (100–199 μg/L). N = 2 studies [35, 38] had iodine deficiency as the reference category (< 100 μg/L), which is why the odds ratios were converted accordingly to sufficient iodine supply as the reference category (Supplement). The pooled OR for iodine deficiency was 1.24 (95% CI [1.16–1.33]) with I^2^ = 0.00 (Fig. 2). The pooled OR for more than adequate iodine supply was 1.01 (95% CI [0.82–1.25]) with I^2^ = 0.79 (Fig. 3). For excessive iodine supply, the pooled OR was 1.00 (95% CI [0.76–1.31]) with an I^2^ = 0.86 (Fig. 4). Due to the small number of studies, it was not possible for us to create a funnel plot.

## Discussion

To the best of our knowledge, this is the first review that systematically analysed the relationship between iodine and the development of thyroid nodules. The majority of the included studies (n = 27 of 31 included study in total) comes from China. To counteract iodine deficiency, iodine has been added to table salt in China since 1996 [55]. Despite the supplementation of iodine in table salt, the prevalence of thyroid nodules has increased since 1996 [38, 55]. A possible explanation for this increase may not only lie in iodine supply but also in improved and more frequent diagnostics. Due to the increased use of high-resolution sonography and modern imaging techniques, thyroid carcinomas and nodules are now detected more frequently than in the past. This makes it difficult to distinguish between a true increase in incidence due to iodine supply effects and a higher detection rate resulting from improved and more frequent diagnostics [58, 59]. Furthermore, it is also difficult to quantify the actual incidence of thyroid cancer. Autopsy studies have already shown that people had latent, asymptomatic, mostly papillary carcinomas that were not detected during their lifetime [60]. It can therefore be assumed that the overall incidence of thyroid cancer is underestimated. The systematic review and meta-analysis by Weng et al. (2017) found no clear correlation between iodine intake and thyroid cancer [56].

N = 23 articles were cross-sectional studies that did not allow any conclusions to be drawn about the causality of iodine for thyroid nodules. There was no RCT collecting prospective data that would allow a more precise investigation of the long-term effects of iodine intake on the development of thyroid nodules. The iodine measurements in the studies were very heterogeneous and therefore difficult to compare. The iodine concentration in urine was measured widely used and categorised into iodine categories defined by the WHO. The concentration of iodine in urine varies considerably throughout the day, depending on recent food and fluid intake. A single urine sample (spot urine) only provides a snapshot and limits the statement about the iodine status of a person. However, spot urines could be sufficient to make statements about the collective iodine status of a region [61].

The results on the correlation between urinary iodine concentration and thyroid nodules are too heterogeneous. Hence, based on the current evidence, it is currently not possible to conclusively clarify whether there is a positive, negative or no association between iodine and thyroid nodules. Although the majority of the mean differences in MUI between the group with thyroid nodules and the group without thyroid nodules were significant, the values of the group with nodules were often in the category of adequate iodine supply. Based on these results, an adequate iodine supply does not appear to have a protective effect against thyroid nodules. The logistic regression analyses also produced contradictory results. Iodine deficiency was a significant risk factor in some studies, but does not in others. In some studies, more than adequate and excessive iodine intake were associated with an increased risk of thyroid nodules, while in others, they appeared to lower the risk. This heterogeneity of the results leaves the hypothesis of a U-shaped relationship, as expressed by Zimmer et al. (2015) [54] and of other studies [14, 30, 43], unconfirmed.

We therefore conducted three meta-analysis to investigate the association between thyroid nodules and iodine deficiency, more than adequate iodine supply and excessive iodine supply. The results showed that iodine deficiency increases the risk of developing thyroid nodules, while more than adequate iodine and excessive iodine supply do not affect the risk. However, the meta-analysis only included n = 7 cross-sectional studies from China, which is why it was not possible to control for a possible publication and cultural bias and the representativeness of the results is limited.

Overall, no evidence-based statement on the relationship between iodine and thyroid nodules is currently possible based on published study results that we found in context of our systematic review.

### Limitations

This review focusing relationship between iodine and thyroid nodules is the first published work that used several databases for the systematic search as well as carried out a supplementary hand search. It can therefore be assumed that all relevant German and English articles on this topic were found, but we cannot rule out the possibility that there are articles in other languages that have addressed this topic. However, one limitation is that we did not create a review protocol in advance and the title-abstract screening was only carried out by one reviewer because of limited human resources in the team. However, two reviewers carried out the full-text screening and a third reviewer was consulted in the event of discrepancies. It must be questioned whether the results of this review and meta-analysis are transferable to Western countries, as the majority of the articles used originate from China. Further research regarding the relationship of iodine and thyroid nodules is needed a) using RCT to verify causal relationships and b) conducting studies in other geographical regions to include cultural/geographical characteristics (such as nutrition, systemic iodisation of drinking water) to test the international transferability of the results). Another limitation concerns the variability in the technical conditions of diagnostic imaging. The sensitivity and specificity of thyroid nodule detection depend significantly on the ultrasound devices and imaging techniques used. Differences in equipment quality, operator expertise, and diagnostic criteria between studies may have influenced the reported prevalence rates of thyroid nodules. Future research should consider standardizing imaging protocols and reporting methods to improve comparability between studies.

## Conclusion

This review and meta-analysis showed that iodine deficiency increases the risk of developing thyroid nodules, while more than adequate or excessive iodine intake had no consistent effects on the risk. However, the results illustrate a considerable heterogeneity of the studies, which makes it difficult to draw evidence-based, causal conclusions. The geographical focus on China and the predominantly cross-sectional design of the studies limit the generalisability of the results. Future research should focus on RCT or prospective long-term cohort studies to better understand the relationship between iodine and thyroid nodules and influencing factors.

## Supplementary Information

Below is the link to the electronic supplementary material.

Supplementary file1 (DOCX 15 kb)

## Data Availability

All data used in this study were extracted from previously published articles included in the systematic review and meta-analysis. These data are publicly available and can be accessed through the original publications, which are cited in the reference list.
