# Supplementary material for: The myth of iodine: A systematic review and meta-analysis on the relationship between iodine and thyroid nodule
Source: J Endocrinol Invest. 2025 May 28;48(8):1693–706. doi: 10.1007/s40618-025-02606-4 (PMC12313809; doi:10.1007/s40618-025-02606-4)
Supplement: Supplementary file 1 — Supplementary file1 (DOCX 15 kb) [file 40618_2025_2606_MOESM1_ESM.docx]

**Supplement - Calculation of odds ratios for the new reference category adequate iodine status**

A = <100 μg/L = iodine deficiency = reference category

B = 100-199 μg/L = adequate iodine status

C = 200-299 μg/L = more than adequate iodine status

D = >299 μg/L = excessive iodine status

Calculation of odds ratios and confidence intervals for iodine deficiency relative to the reference category of sufficient iodine supply

OR_A vs B_ = 1/OR_B vs A_

Calculation of odds ratios and confidence intervals for more than adequate iodine status / excessive iodine status in relation to the reference category of adequate iodine status

adequate iodine status

log(OR)_C vs A_ = ln(OR)_C vs A_

log(OR)_B vs A_ = ln(OR)_B vs A_

log(OR)_C vs B_ = log(OR)_C vs A_ - log(OR)_B vs A_

SE_ln(OR C vs A)_ = (ln (CI Upper)_C vs A_ – ln (CI Lower)_C vs A_) / 2 * 1.96

SE_ln(OR B vs A)_ = (ln (CI Upper)_B vs A_ – ln (CI Lower)_B vs A_) / 2 * 1.96

SE_ln(OR C vs B)_ = √ ((SE_ln(OR C vs A)_)^2^ + (SE_ln(OR B vs A)_)^2^)

CI_ln(OR C vs A)_ = log(OR)_C vs B_ ± 1.96 * SE_ln(OR C vs B)_

CI = [e^CI_Lower^ ; e^CI_Upper^]

OR_C vs B_ = e^logOR C vs B^

excessive iodine status

log(OR)_D vs A_ = ln(OR)_D vs A_

log(OR)_B vs A_ = ln(OR)_B vs A_

log(OR)_D vs B_ = log(OR)_D vs A_ - log(OR)_B vs A_

SE_ln(OR D vs A)_ = (ln (CI Upper)_D vs A_ – ln (CI Lower)_D vs A_) / 2 * 1.96

SE_ln(OR B vs A)_ = (ln (CI Upper)_B vs A_ – ln (CI Lower)_B vs A_) / 2 * 1.96

SE_ln(OR D vs B)_ = √ ((SE_ln(OR D vs A)_)^2^ + (SE_ln(OR B vs A)_)^2^)

CI_ln(OR D vs A)_ = log(OR)_D vs B_ ± 1.96 * SE_ln(OR D vs B)_

CI = [e^CI_Lower^ ; e^CI_Upper^]

OR_D vs B_ = e^logOR D vs B^
